# Identification of PLCG2 activators for the treatment of Alzheimer’s disease

**DOI:** 10.1002/alz.084240

**Published:** 2025-01-09

**Authors:** Brent Clayton, Steven M Massey, Daniel E Beck, Karson S Putt, Tada Utsuki, Ramya Visvanathan, Andrew D. Mesecar, Emma K Lendy, Bridget L Kaiser, Shaoyou Chu, Emily R Mason, Bruce T. Lamb, Alan D. Palkowitz, Timothy I. Richardson

**Affiliations:** ^1^ Indiana University School of Medicine, Indianapolis, IN USA; ^2^ Purdue, West Lafayette, IN USA; ^3^ Indiana Biosciences Research Institute, Indianapolis, IN USA

## Abstract

**Background:**

The goal of the TREAT‐AD Center is to enable drug discovery by developing assays and providing tool compounds for novel and emerging targets. The role of microglia in neuroinflammation has been implicated in the pathogenesis of Alzheimer’s disease (AD). Genome‐wide association studies, whole genome sequencing, and gene‐expression network analyses comparing normal to AD brain have identified risk and protective variants in genes essential to microglial function. among them. The P522R variant of phospholipase C gamma2 (PLCγ2) is associated with reduced risk for AD and has been characterized as a functional hypermorph. Carriers of P522R with mild cognitive impairment exhibited a slower cognitive decline rate. Conversely the M28L variant increases risk. Therefore, activation of the protein PLCγ2 with small molecules has been proposed as a therapeutic strategy to reduce the rate of disease progression and cognitive decline in AD patients.

**Method:**

We performed a high‐throughput screen using affinity selection mass spectrometry (ASMS) to identify novel small molecules that bind to the full‐length protein PLCγ2. A Cellular Thermal Shift Assay (CETSA) was developed to confirm target engagement in cells. A liposomal‐based, fluorogenic reporter biochemical assay was implemented to evaluate activity of the enzyme. A high‐content imaging assay measuring phagocytosis, cell number, and nuclear intensity was carried out using the BV2 and HMC3 cell lines to characterize cellular pharmacology and cytotoxicity. Structure activity relationship (SAR) studies were performed to synthesize analogs and optimize for binding and cellular pharmacology. Optimized compounds have been studied *in vivo* to assess pharmacokinetic properties and drug likeness.

**Result:**

Novel PLCγ2 activators have been discovered and preliminary optimization has been completed. These compounds have shown positive results for target engagement, biochemical activity, and cellular pharmacology. *In silico* predictions indicated the molecule structures are suitable CNS drug discovery program starting points.

**Conclusion:**

Activation of PLCγ2 is a novel therapeutic strategy for treatment of AD. We identified structurally distinct molecular scaffolds capable of enzyme activation and cellular activity. Recommendations for use of probe molecules in target validation studies and the development of lead‐like molecules for clinical studies will be made.

